# Hydrocarbon Patterns and Mating Behaviour in Populations of *Drosophila yakuba*

**DOI:** 10.3390/insects6040897

**Published:** 2015-10-28

**Authors:** Béatrice Denis, Arnaud Le Rouzic, Claude Wicker-Thomas

**Affiliations:** Laboratoire Évolution, Génomes, Comportement et Écologie, CNRS, IRD, Université Paris-Sud, Université Paris-Saclay, Gif-sur-Yvette F-91198, France; E-Mails: beatrice.denis@egce.cnrs-gif.fr (B.D.); Arnaud.Le-Rouzic@egce.cnrs-gif.fr (A.L.R.)

**Keywords:** *Drosophila yakuba*, cuticular hydrocarbons, mating behavior, reproductive isolation

## Abstract

*Drosophila yakuba* is widespread in Africa. Here we compare the cuticular hydrocarbon (CHC) profiles and mating behavior of mainland (Kounden, Cameroon) and island (Mayotte, Sao-Tome, Bioko) populations. The strains each had different CHC profiles: Bioko and Kounden were the most similar, while Mayotte and Sao-Tome contained significant amounts of 7-heptacosene. The CHC profile of the Sao-Tome population differed the most, with half the 7-tricosene of the other populations and more 7-heptacosene and 7-nonacosene. We also studied the characteristics of the mating behavior of the four strains: copulation duration was similar but latency times were higher in Mayotte and Sao-Tome populations. We found partial reproductive isolation between populations, especially in male-choice experiments with Sao-Tome females.

## 1. Introduction

Long-chain cuticular hydrocarbons (CHCs) are fatty acid-derived hydrocarbons present on the *Drosophila* cuticle. They protect against desiccation and stimulate courtship [1,2]. CHCs have been thoroughly characterized in the nine species of *melanogaster* subgroup, and there is evidence that CHCs with at least one double bond in position (*Z*)-7 function as pheromones [3].

In the *melanogaster* subgroup the chemical composition of CHCs is quite similar in males regardless of the species, with very abundant monoenes in C23 and C25 (7-tricosene (7-T) and 7-pentacosene (7-P)), except in *D. erecta* [4]. CHC composition in females is species dependent, with a CHC sexual dimorphism in three species: *D. melanogaster*, *D. sechellia* and *D. erecta*. The remaining six species have similar CHC profiles in males and females: *D. yakuba*, *D. santomea*, *D. teissieri*, *D. orena*, *D. mauritiana* and *D. simulans* [4].

Female pheromones contribute to reproductive isolation between *D. melanogaster* and *D. simulans* [5], *D. sechellia* and *D. mauritiana* [6], and *D. sechellia* and *D. simulans* [7]. Within *D. melanogaster*, a female CHC polymorphism is linked to the geographic origin of two populations, characterized by 5,9-dienes in African and Caribbean populations and 7,11-dienes in all other populations [8]. This CHC polymorphism is involved in reproductive isolation [9]. Male pheromones also play a role in reproductive isolation between *D. santomea* and *D. yakuba* [10], and between populations of *D. melanogaster* [11] and *D. simulans* [12].

All the *melanogaster* subgroup species originated in the African tropics. Some of them are cosmopolitan (*D. melanogaster*, *D. simulans*), some are island endemics (*D. sechellia*, *D. santomea*, *D. mauritiana*), or more or less confined to a restricted area of the African mainland (*D. teissieri*, *D. orena*). One (*D. erecta*) is specialized on *Pandanus* fruits. The last species (*D. yakuba*) is broadly distributed across tropical sub-Saharan Africa [13]. It is also abundant on the islands near the continent, such as Madagascar [14].

Because of its widespread distribution, we wondered whether *D. yakuba* might show some geographic differentiation, leading to reproductive isolation between different populations. To address this question, we performed pheromone analyses and mating tests on one population from mainland Cameroon and three populations confined to different African islands.

## 2. Experimental Section

### 2.1. Drosophila Strains

*D. yakuba* flies were maintained on standard yeast/cornmeal/agar medium in a 12-h-light/12-h-dark cycle at 25 °C. We used four strains, issued from isofemale lines (given and collected by J. David in 2013). One was from the mainland in Cameroon: Kounden (K) (latitude 5°41′, longitude 10°40′). Three were from different islands: Bioko (B) (latitude 3°30′, longitude 8°42′); Sao-Tome (S) from the west coast of Africa (latitude 0°20′, longitude 6°43′ S, collected at an altitude of about 1200 m); and Mayotte (M) from southeast Africa (latitude 12°46′, longitude 45°13′). All strains were maintained in the laboratory for two years. Flies were sexed, isolated under carbon dioxide anesthesia within 2 h of imaginal emergence, and kept in sex-specific groups of 10 in fresh food vials until testing. Five-day-old flies were used for the experiments.

### 2.2. Cuticular Hydrocarbon Extraction and Analyses

CHCs were extracted from individual 5-day-old flies by washing them for 5 min in 100 µL of heptane containing 500 ng hexacosane (C26) as an internal standard. The fly was then removed from the vial and 5 µL of each sample was injected into a Perichrom Pr200 gas chromatograph with hydrogen as the carrier gas, using a split injector (split ratio 40:1). The column was a 25 m long BP-1 that had an internal diameter of 220 μm and 0.1 μm film thickness (SGE). The oven temperature started at 180 °C then ramped at 3 °C/min to 300 °C, for a total run of 40 min. The flame ionization detector was set at 260 °C [12]. CHCs were identified by GC-MS and then by their retention times. We used Winilab III software (version 04.06, Perichrom, Saulx-les-Chartreux, Île-de-France, France, 1999) to carry out the peak integration of hydrocarbon data. We quantified 14 CHCs for each male, with a chain length ranging between 23 and 29 carbons. Data are presented as means ± SEM (*n* = 10 for all measurements).

### 2.3. Mating Experiments

All tests were run in the morning, between 9 and 12 a.m. under dim electric light in a temperature-controlled room. For the mating tests, we used a watchglass of 28 mm diameter and 5 mm internal height glass placed it on a glass plate as an observation chamber. We introduced a female into the observation chamber and left her for 1 min before introducing a male. We recorded copulation latency (time from introduction of the male into the observation chamber to copulation) and the duration of copulation. Individuals that did not mate within 45 min were not used in the analysis of copulation latency (*n* ≥ 50 for all tests).

We used rearing tubes (2 cm diameter; 9 cm length, filled with rearing medium to a height of 2 cm) for choice tests, to allow the flies to access the food and to give females sufficient room to escape from males. We cut a small portion of one wing (alternately right or left) of all the flies to allow us to differentiate flies of different strains in mate-choice tests. For female-choice and male-choice tests, a single female (male) was transferred to a fresh food vial together with two males (females) from different strains. The trial ended once the female had copulated with one of the males.

For multiple-choice tests, two males and two females (one male and one female of each strain) were introduced into a fresh food vial. The trial ended when the first copulation occurred. We did the multiple-choice tests because they are more sensitive at detecting assortative mating than are no-choice tests and they allow both male and female choice to contribute to assortative mating [15]. We carried out all assays for female- and multiple-choice tests until 30 copulations had occurred or for one hour, whichever came first. For statistical purposes, we also recorded the number of flies that did not mate.

### 2.4. Statistics

All statistical analyses were performed using R version 3.2.1 (R Foundation for Statistical Computing, Vienna, Austria, 2015).

#### 2.4.1. CHC

CHC data were normalized relative to 2-methylhexacosane (Me26), which was proportionally the least variable compound. The analysis was thus performed on the logarithm of the ratio between measured abundance and Me26. We replaced measurements of 0.0 with 0.1 (close to the detection limit with our protocol) before log transformation.

The effects of sex, population, and the sex-by-population interaction were tested using a two-way multivariate ANOVA (*manova* function in R), and *p*-values were corrected for multiple tests (Holm-Bonferroni correction). When the population factor appeared to be significant in the ANOVA, we used post-hoc analyses to compare pairwise differences among populations (Tukey HSD test). In addition, we used principal component analysis to evaluate abundances (function *prcomp* in R).

#### 2.4.2. Mating Experiments

In the 1 + 2 mating experiments (1 female/2 males and 1 male/2 females), we considered three categories: Homogeneous crosses, heterogeneous crosses, and no mating. We used the *p*-value of a two-tailed binomial test (twice the result of the function *pnorm* in R) to quantify departure from the random mating hypothesis (H_0A_: equal homogeneous and heterogeneous crosses). Multiple testing-corrected *p*-values (Holm-Bonferroni correction) are indicated as p*. The specific influence of male/female population on the homogeneous/heterogeneous cross ratio was investigated by running binomial generalized linear models (GLM):

Prob(homogeneous)_mf_ = *g*^−1^ (µ + x_m_ + y_f_ + z_mf_), (1)

where *m* and *f* are the population of the introduced male/female, µ is the reference probability (intercept, see below), x_m_ and y_f_ describe the effects the male and female population and z_mf_ is the interaction between both populations. The link function *g* was the default logit function. Conveniently, depending on the hypothesis being tested, the intercept could be set to µ = 0 (in which case, the model tests for departure from the random mating hypothesis) or it could be computed arbitrarily as the average of the first experiment (in which case the model estimates differences between experiments). The general significance of the factors was assessed with an analysis of deviance (function *anova.glm* in R), which determines the most parsimonous models by adding factors one by one (forward model selection) and performing likelihood ratio tests. As the factor order matters in such models, we made sure to introduce the factors in the following order: (i) the population of the tested individual (female in the 1 female/2 males test; male in the 1 male/2 females test); (ii) the population of the heterogeneous cross (male and female, respectively); and (iii) the interaction between the two.

The same setting was used to test the influence of male/female populations on the proportion of trials in which there were no mating events (in which case the intercept µ was always an estimate, since the 50/50 hypothesis has no meaning). Finally, the same factors were considered for the analysis of copulation latency (linear model after log-transformation).

In the 2 + 2 experiments, four categories were defined: homogeneous for population 1, homogeneous for population 2, heterogeneous, and no mating. For each experiment, we tested three hypotheses: (1) equal homogeneous and heterogeneous crosses (H_0A_); (2) equal numbers of each kind of homogeneous crosses (H_0B_); and (3) random mating (H_0C_), in which 25% of the mating were homogeneous crosses of each type and 50% were heterogeneous crosses. For H_0C_, for convenience, the multinomial *p*-value was computed by Monte-Carlo simulations (*simulate.p.value* option in the *chisq.test* function in R).

## 3. Results

### 3.1. Cuticular Hydrocarbons

**Figure 1:**
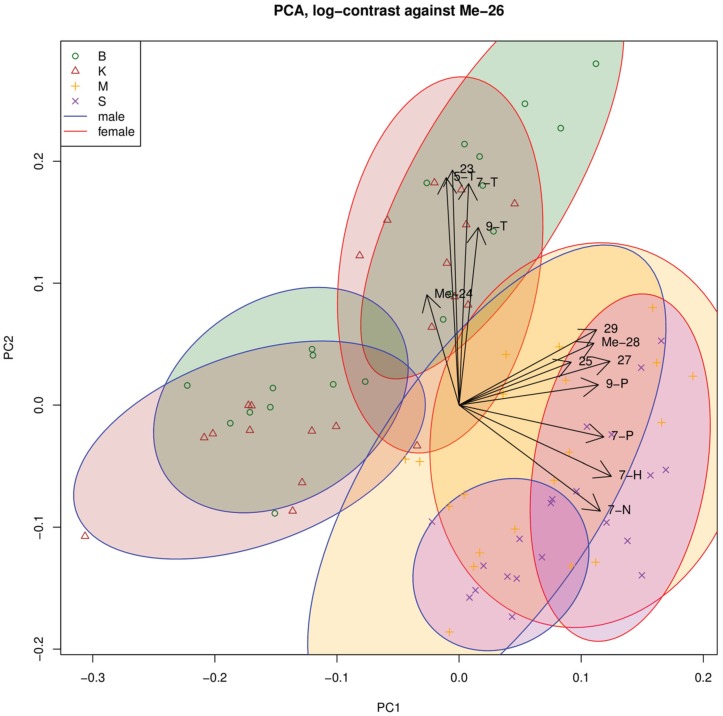
Principal component analysis of CHC compositions in the different populations. Bioko (B), Kounden (K), Mayotte (M), Sao-Tome (S).

**Figure 2:**
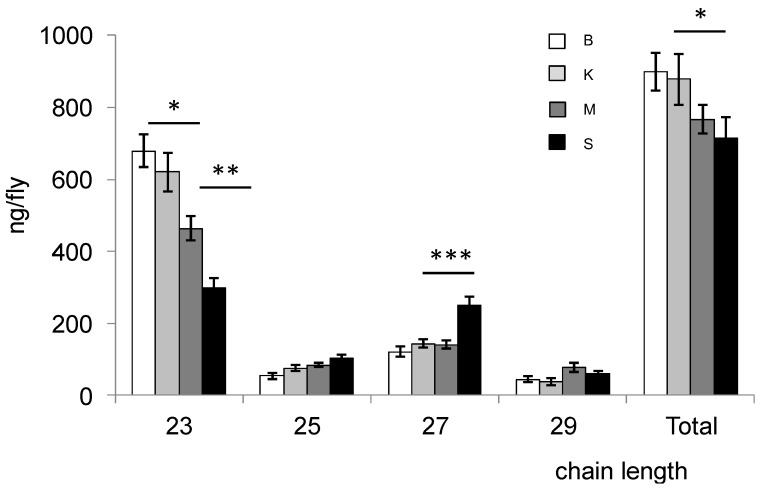

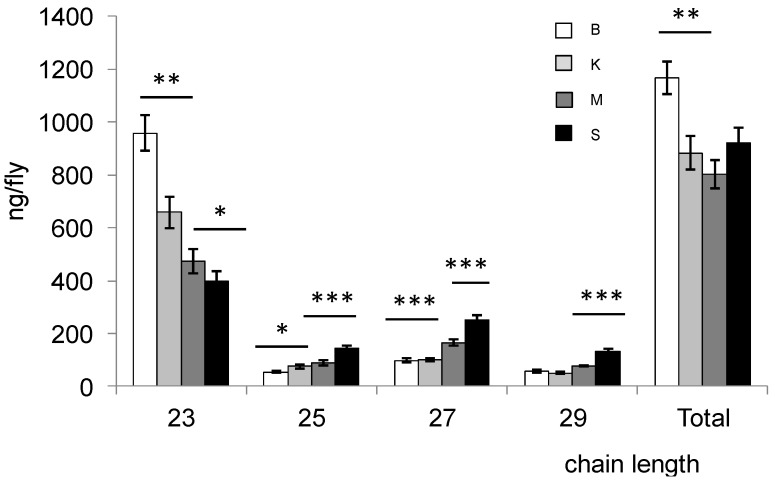
Amount of hydrocarbons of different lengths extracted from male (**top**) and female (**bottom**) *D. yakuba* flies from Bioko (B), Kounden (K), Mayotte (M) and Sao-Tome (S) populations. Total: C23 to C29; Each bar represents mean ± SEM (*n* = 10). *, ** and *** above bars indicate significant differences (one-way ANOVA, *p* = 0.05, 0.01 and 0.001, respectively) between means.

CHC composition for each strain is reported in Table 1 and Table 2. Principal component analysis of the CHC composition in each population separated the values according to (7-H, 7-P, 9-P, 7-N, 25:0, 27:0, 29:0, Me-28) for the first component and (7-T, 9-T, 23:0) for the second component (Figure 1). As the second component contains the CHCs in C23, we wondered whether there was a difference in CHC chain length among the populations (Figure 2). Bioko and Kounden males had the most CHCs (17% more than Mayotte and Sao-Tome males). The difference in C23 compounds was particularly large (8.6%, 31.6% and 55.8% less in Kounden, Mayotte, Sao-Tome males than in Bioko). Sao-Tome males had also two times more C27 than males from the other populations.

**Table 1:** Male CHC profiles of four strains of *D. yakuba*.

CHC	Bioko	Kounden	Mayotte	Sao-Tome
23–29 tot	898.9 ± 52.1	877.8 ± 71.8	766.3 ± 125.6	714.7 ± 58.4
9-T	2.17 ± 0.17	5.27 ± 1.04	4.36 ± 1.04	3.69 ± 0.89
7-T	57.51 ± 2.84	48.56 ± 1.97	45.95 ± 1.74	27.90 ± 1.82
5-T	1.20 ± 0.09	1.61 ± 0.14	0.94 ± 0.13	1.04 ± 0.12
23:0	14.48 ± 0.73	14.41 ± 0.63	8.21 ± 0.58	9.13 ± 0.54
Me-24	0.98 ± 0.12	1.19 ± 0.11	0.53 ± 0.17	0.92 ± 0.10
9-P	0.36 ± 0.06	0.67 ± 0.08	1.43 ± 0.13	1.52 ± 0.11
7-P	1.19 ± 0.14	2.82 ± 0.25	4.84 ± 0.40	5.91 ± 0.27
25:0	3.43 ± 0.80	3.79 ± 0.43	4.24 ± 0.48	5.88 ± 0.47
Me-26	10.61 ± 0.85	14.37 ± 0.53	11.54 ± 1.04	14.04 ± 0.48
7-H	0.49 ± 0.06	0.19 ± 0.05	3.66 ± 0.49	15.90 ± 1.41
27:0	2.44 ± 0.66	1.97 ± 0.39	3.67 ± 0.35	4.64 ± 0.34
Me-28	4.34 ± 0.61	3.90 ± 0.93	8.73 ± 1.09	4.23 ± 0.47
7-N	0.0 ± 0.0	0.04 ± 0.04	0.28 ± 0.07	3.84 ± 0.61
29:0	0.42 ± 0.12	0.49 ± 0.12	0.81 ± 0.12	0.66 ± 0.11

CHC identities are given in the first column. The first line gives the total quantity (ng/fly) of CHCs produced by 10, 5-day-old males at 25 °C. The other lines give the CHC percentages (m ± SEM).

**Table 2:** Female CHC profiles of four strains of *D. yakuba*.

CHC	Bioko	Kounden	Mayotte	Sao-Tome
23–29 tot	1168.8 ± 61.4	883.2 ± 64.7	803.9 ± 53.6	921.1 ± 56.0
9-T	3.08 ± 0.26	9.69 ± 1.34	3.89 ± 0.99	2.91 ± 0.62
7-T	58.94 ± 1.61	46.46 ± 2.62	46.11 ± 1.78	28.13 ± 2.18
5-T	1.44 ± 0.14	2.41 ± 0.44	0.74 ± 0.10	0.85 ± 0.14
23:0	17.65 ± 0.42	14.44 ± 0.92	6.69 ± 0.77	10.28 ± 0.84
Me-24	0.41 ± 0.05	0.97 ± 0.18	0.41 ± 0.05	0.82 ± 0.17
9-P	0.43 ± 0.04	1.28 ± 0.19	2.30 ± 0.30	2.01 ± 0.09
7-P	1.09 ± 0.06	2.70 ± 0.35	5.29 ± 0.60	7.50 ± 0.42
25:0	2.92 ± 0.56	3.56 ± 0.43	3.01 ± 0.29	5.11 ± 0.41
Me-26	4.82 ± 0.57	8.14 ± 0.83	8.14 ± 0.61	9.13 ± 0.47
7-H	0.77 ± 0.06	0.60 ± 0.11	6.48 ± 0.97	11.98 ± 0.64
27:0	3.10 ± 0.39	3.00 ± 0.35	6.57 ± 0.81	6.11 ± 0.63
Me-28	4.26 ± 0.51	5.29 ± 0.53	8.01 ± 0.50	7.22 ± 0.87
7-N	0.04 ± 0.02	0.04 ± 0.03	0.62 ± 0.13	5.56 ± 0.53
29:0	0.77 ± 0.10	0.56 ± 0.09	1.19 ± 0.15	1.56 ± 0.21

CHC identities are given in the first column. The first line gives the total quantity (ng/fly) of CHCs produced by 10, 5-day-old females at 25 °C. The other lines give the CHC percentages (m ± SEM).

Females from Bioko had the most CHCs (35% more than females from the other populations). There was also a large discrepancy in the amount of C23 (31.3%, 50.7% and 58.6% less in Kounden, Mayotte, Sao-Tome females than in Bioko). Sao-Tome females had the most C25, C27 and C29 (161.9%, 153.6% and 129.4% more than Bioko females).

**Figure 3:**
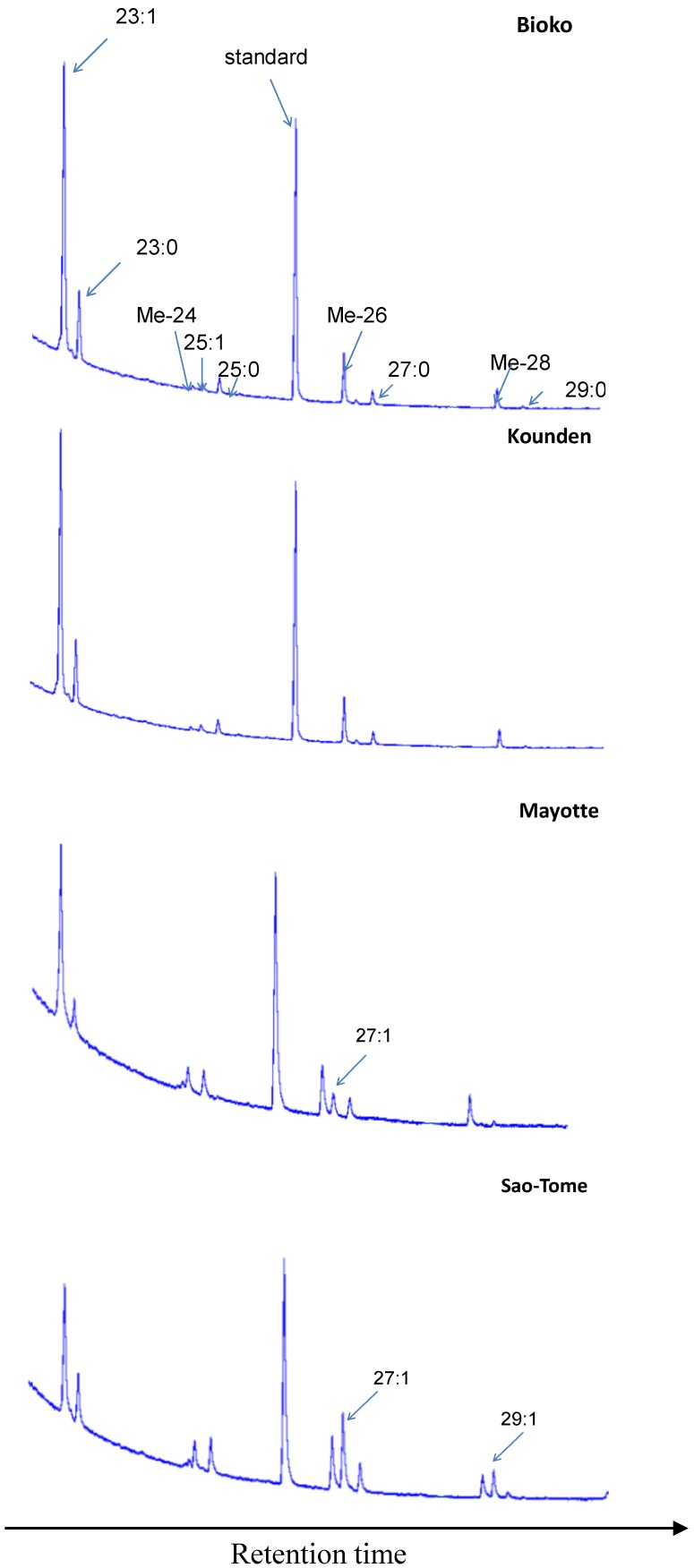
Gas chromatograms of *D. yakuba* males. 23:1 (tricosenes), 23:0 (tricosane), Me-24 (2-methyltetracosane), 25:1 (pentacosenes), 25:0 (pentacosane), standard (hexacosane), Me-26 (2-methylhexacosane), 27:1 (7-heptacosene), 27:0 (heptacosane), Me-28 (2-methyloctacosane), 29:1 (7-nonacosene), 29:0 (nonacosane).

Figure 3 gives typical examples of male CHC chromatograms. The largest differences concerned 7-H, and 7-N:7-H represents about 16% of the CHCs in Sao-Tome, and only 0.5% in Bioko. 7-N represents 4% of the CHCs in Sao-Tome, but is barely detectable in Kounden and Mayotte and is absent in Bioko.

### 3.2. Mating Behavior and Mate Discrimination between Strains

Bioko males more often exhibited courtship and eventually mated than did males from the other populations. Around 50%, of Kounden, Mayotte and Sao-Tome males mated—about 28% less than Bioko (Table 3). Latency times were high and somewhat higher for Mayotte and Sao-Tome (around 24 min) than for Kounden (around 18 min). Copulation duration did not differ significantly among populations.

**Table 3:** Mating behavior parameters of *D. yakuba* strains.

Population	Total Number	Copulation Percentage	*n*	Copulation Latency	Duration of Copulation
**Bioko**	50	70	35	21.56 ± 1.14 ^ab^	41.50 ± 1.35 ^a^
**Kounden**	51	60.78	31	18.03 ± 1.07 ^b^	38.81 ± 1.72 ^a^
**Mayotte**	54	51.85	28	24.14 ± 1.61 ^a^	36.19 ± 2.15 ^a^
**Sao-Tome**	62	50	31	24.53 ± 0.86 ^a^	40.95 ± 1.67 ^a^

Copulation latency and copulation duration (in min) are expressed as m ± SEM. In each column, values followed by different letters are significantly different at the *p* = 0.05 level.

All mating behavior experiments showed a significant departure from random mating, suggesting partial reproductive isolation (253 homogeneous pairs *vs.* 177 heterogeneous, *p*-value < 0.001 in the 1 female/2 male experiment; 219 *vs.* 145 pairs, *p*-value < 0.001 in the 1 male/2 female experiment; and 126 *vs.* 67 pairs, *p*-value < 0.001 in the 2 males/2 females experiment) (Table 4, Table 5 and Table 6).

**Table 4:** Female-choice experiment (1 female/2 males). The three categories were: homogeneous crosses, (Hom), heterogeneous crosses (Het), and no mating. Departure from the random mating hypothesis (H_0A_: as many homogeneous as heterogeneous crosses) was quantified using the bilateral binomial *p*-value. Multiple testing-corrected *p*-values (Holm-Bonferroni correction) are indicated as p*; B: Bioko; K: Kounden; M: Mayotte; S: Sao-Tome.

Female	Male	Hom	Het	No Mating	*p* (H_0A_)	p* (H_0A_)
B	K	21	17	8	0.627	1.000
B	M	20	14	3	0.392	1.000
B	S	17	20	5	0.743	1.000
K	B	25	27	2	0.890	1.000
K	M	18	20	9	0.871	1.000
K	S	21	11	18	0.110	0.992
M	B	22	13	8	0.175	1.000
M	K	23	11	18	0.058	0.576
M	S	17	13	14	0.585	1.000
S	B	23	7	18	0.005	0.057
S	K	28	7	11	0.001	0.006
S	M	18	17	11	1.000	1.000

**Table 5:** Male choice experiment (1 male/2 females). See Table 4 for descriptions of categories.

Male	Female	Hom	Het	No Mating	*p* (H_0A_)	p* (H_0A_)
B	K	16	14	3	0.856	1.000
B	M	21	10	6	0.071	0.637
B	S	23	8	19	0.011	0.117
K	B	10	9	6	1.000	1.000
K	M	25	20	17	0.551	1.000
K	S	28	8	19	0.001	0.014
M	B	16	15	3	1.000	1.000
M	K	9	11	6	0.824	1.000
M	S	13	14	10	1.000	1.000
S	B	17	16	7	1.000	1.000
S	K	17	9	4	0.169	1.000
S	M	24	11	9	0.041	0.410

**Table 6:** Multiple choice experiment (2 males/2 females). The four categories were: Hom 1: Homogeneous for population 1 (Hom 1), homogeneous for population 2 (Hom 2), heterogeneous (Het), and no mating (no). For each experiment, we tested three hypotheses (H_0A_: equal homogeneous and heterogeneous crosses, H_0B_: equal homogeneous crosses of each kind, H_0C_: random mating, which means 25% of each homogeneous cross and 50% heterogeneous crosses).

Pop 1	Pop 2	Hom 1	Hom 2	Het	No	*p* (H_0A_)	p* (H_0B_)	*p* (H_0B_)	p* (H_0B_)	*p* (H_0C_)	p* (H_0C_)
B	K	8	5	18	5	0.482	0.964	0.569	1.000	0.498	0.498
B	M	14	9	12	5	0.097	0.388	0.417	1.000	0.102	0.408
K	M	13	15	10	2	**0.003**	**0.018**	0.843	1.000	0.016	0.096
B	S	4	14	17	2	1.000	1.000	0.028	0.168	0.066	0.330
K	S	10	12	12	6	0.114	0.388	0.831	1.000	0.222	0.444
M	S	10	12	11	6	0.073	0.365	0.836	1.000	0.146	0.438

In the female choice experiment (Table 4), the analysis of deviance identified a significant effect of the female population on the proportion of homogeneous pairs (*p* = 0.04), but no effect of the male population (*p* = 0.32). The GLM with no intercept (test for the departure from random mating) attributes this effect to females from the Mayotte population (*p* = 0.01) and from the Sao-Tome population (*p* < 0.001), while Bioko and Kounden females seem to mate randomly (*p* = 0.50 and *p* = 0.59, respectively). There was also a significant female effect on the proportion of non-mating events (*p* = 0.002 in the analysis of deviance). Females from populations Bioko and Kounden mated more often than females from populations Mayotte and Sao-Tome. We found no significant effect from population on copulation latency for either females (*p* = 0.06) or males (*p* = 0.64).

In the male choice experiment, we found no evidence of a differential effect of male (*p* = 0.13 in the analysis of deviance) or female (*p* = 0.16) population on the proportion of homogeneous crosses, although some specific crosses (such as Kounden male × Sao-Tome female) departed significantly from random mating hypothesis on their own. In contrast, there was a significant effect of female population on the proportion of pairs in which there was no mating (*p* = 0.009)—an effect that was not observed for males (*p* = 0.11). More specifically, the GLM identified a significant departure of females from the Sao-Tome population (*p* = 0.0028) compared to the Bioko population, which was taken arbitrarily as a reference. This strongly suggests that the presence of a Sao-Tome female in the experiment prevented any male from another population from mating with the other female. We found no significant effect of male (*p* = 0.46) or female (*p* = 0.05) population on copulation latency.

In the multiple-choice test, testing for random mating is more complicated as there can be deviations from the 1:1 ratio of homogeneous/heterogeneous crosses and/or uneven frequencies of homogeneous crosses. A binomial GLM with no intercept showed that only the Mayotte population had a significant influence on the excess of homogeneous crosses (*p* = 0.002). In contrast, Sao-Tome and Bioko populations were the only ones driving a significant deviation from an even representation of both kinds of homogeneous pairs (4 Bioko-Bioko pairs *vs.* 14 Sao-Tome-Sao-Tome pairs, *p* = 0.027). Sao-Tome population showed a general trend towards being preferentially associated with such deviations, but this average effect was not significant (*p* = 0.057). We did not detect any population effect on copulation latency or on the proportion of pairs in which there were no mating events.

## 4. Discussion

In the *melanogaster* subgroup, only three species show a wide distribution: the two cosmopolitan species *D. melanogaster* and *D. simulans*, and *D. yakuba*, which is abundant on the African mainland and islands [13]. A genome study comparing *D. yakuba* and *D. simulans* found more genetic variation in *D. yakuba*, which displays 1.5 times as many duplications and twice as many deletions than *D. simulans* [16]. This rapid flux of duplications and deletions is expected to have significant effects on adaptation and the evolution of novel traits [17]. In fact, we found different CHC profiles between two island populations, Mayotte and Sao-Tome.

### 4.1. Cuticular Hydrocarbons

Both males and females of most species of the *melanogaster* subgoup have the same CHC profiles, with the exception of three species: *D. melanogaster*, *D. sechellia* and *D. simulans* [4]. The six monomorphic species all have very similar CHC profiles, characterized by a high proportion of 7-tricosene, which indicates equivalent biosynthesis pathways. Bioko and Kounden populations of *D. yakuba* have similar CHC profiles to all other Drosophila species. However, the CHC profiles of the Mayotte and Sao-Tome populations are quite different. The CHC profile of the Mayotte population includes less tricosene and tricosane and has 7-heptacosene and 7-nonacosene, which are both nearly absent from the monomorphic species and from the other *D. yakuba* strains. The CHC profile of the Sao-Tome population, which is located on another island 400 km from Bioko, is characterized by low tricosene and very high heptacosene and nonacosene.

Hydrocarbon biosynthesis originates from fatty acids that are desaturated, elongated and eventually decarboxylated. In *D. melanogaster*, we have shown an elongase, *eloF*, is responsible for the formation of alkenes and alkanes in C27 and C29. When inhibited by RNAi, the hydrocarbons in C27 and C29 are replaced by smaller ones, in C23 and C25 [18]. A differential expression of this elongase in the *D. yakuba* populations might account for these differing CHC profiles.

The Sao-Tome strain used in this study was collected at about 1200 m altitude in a zone where hybrids between *D. santomea* and *D. yakuba* can be found at a low frequency (~1%) [19,20]. *D. yakuba* is also found at low elevation. In Sao-Tome, the *D. yakuba* strains occurring at high altitude are thought to be more ancient that the ones occurring at low altitude, because *D. santomea*, which diverged from a common ancestor with *D. yakuba* 400,000 years ago [21,22], inhabits mountain forests above 1150 m. The occurrence of *D. yakuba* throughout lowland Sao-Tome may be the result of a recent secondary invasion [14]. Mas and Jallon [10], analysing the CHCs from a *D. yakuba* strain collected at low altitude, showed a CHC profile similar to that of Bioko and Kounden. These results strengthen the hypothesis that *D. yakuba* flies at lowland sites were recently introduced.

### 4.2. Mating Behavior and Reproductive Isolation

Copulation latencies were high for all strains, compared to others reported in the literature [10,23] (about 15 min). One study [10] put three females and three males together and recorded the time to first copulation, which explains why they measured shorter copulation latencies. In another study [23], flies were observed under watchglasses containing food medium, which may also have enhanced mate attraction [3]. Copulation latencies measured in our experiments are similar those measured in another study that used the same protocol that we used [24]. We observed a long copulation duration (40 min) compared to *D. melanogaster* (about 20 min) under the same conditions. An even longer copulation duration has been reported in *D. yakuba* (60 min for the Abidjan strain). Copulation in most *Drosophila* species lasts about 30 min [24].

Courtship behavior of Sao-Tome females seems to differ from that of the other strains: they take longer before they begin to copulate and the percentage of non-mating females is higher. When put with mates from other strains in both the male-choice and female-choice experiments, the percentage of trials in which no flies mate is high, suggesting that Sao-Tome females prevent all males from mating. Other experiments are still needed to study whether this behavior is due to their unique CHC profile.

Although the proportion of homogeneous matings was generally higher than the proportion of heterogeneous ones, we lacked statistical power to demonstrate significant reproductive isolation between strains, except between Kounden and Mayotte (multiple-choice tests). In previous unpublished experiments, we measured mate choice using the small watchglass apparatus used here to measure courtship behaviour, and observed strong reproductive isolation between strains. We cannot totally explain this discrepancy. In nature, flies have continual access to food, which has an attractive role and is also important for mating behavior. The device used in this study is more appropriate for choice experiments because flies can smell food, eat, fly and escape from an undesired partner. In some species (*D. pseudoobscura* and *D. persimilis*), courtship occurs under five seconds and mating within the first two minutes both in the laboratory and the field. In this case, no-choice designs are reflecting nature better than choice-designs [25]. In contrast, copulation latency is high in *D. yakuba* and in these conditions, choice-designs seem more appropriate than no-choice ones because females have the time to choose a mate between different males. However, we cannot prevent any bias resulting from the different copulation latencies within *D. yakuba* populations. We have used one-choice and multiple-choice experiments. Although some authors suggest that multiple-choice designs are more realistic because flies congregate before mating on food, this does not seem to be justified, because very little is known on the circumstances of mating, the density and sex-ratio of flies in the mating habitat [26].

## 5. Conclusions

We were able to demonstrate a peculiar mating behavior within the Sao-Tome population. As CHC profiles differ between the strains, it is tempting to emphasize the role of CHCs in mate choice. Although CHCs contribute to reproductive isolation in most species within the melanogaster subgroup, other factors, such as courtship songs, could play a meaningful role [27]. The *melanogaster* subgroup species exhibit species-specific songs [28]. Sexual isolation between *D. santomea* and *D. yakuba* is largely due to discrimination against *D. yakuba* males by *D. santomea* females [26]. *D. santomea* males differ from from *D. yakuba* both by the presence of heneicosane in their CHC profile and by their courtship song [29]. *D. santomea* males also have a unique courtship behaviour: they rotate their wing blades to face forward and then back (rowing) [30]. The evolution of courtship song and behavior may have played a role in the strong reproductive isolation between these two species. Likewise, chemical cues associated with acoustic and behavioral ones may have evolved to differentiate *D. yakuba* populations. The rapid genome evolution described in isogenic lines from *D. yakuba* [13] might drive reproductive isolation in the future.
